# Biomarkers in Alzheimer’s disease: role in early and differential diagnosis and recognition of atypical variants

**DOI:** 10.1186/s13195-023-01314-6

**Published:** 2023-10-13

**Authors:** Bruno Dubois, Christine A. F. von Arnim, Nerida Burnie, Sasha Bozeat, Jeffrey Cummings

**Affiliations:** 1 Assistance Publique-Hôpitaux de Paris (AP-HP), Memory and Alzheimer’s Disease Institute, Sorbonne University, Paris, France; 2 Brain Institute, Sorbonne University, Paris, France; 3 Department of Geriatrics, University Medical Center Göttingen, Göttingen, Germany; 4 General Practice, South West London CCG, London, UK; 5 London Dementia Clinical Network, London, UK; 6 F. Hoffmann-La Roche AG, Basel, Switzerland; 7 Chambers-Grundy Center for Transformative Neuroscience, Pam Quirk Brain Health and Biomarker Laboratory, Department of Brain Health, School of Integrated Health Sciences, University of Nevada Las Vegas, Las Vegas, NV USA

**Keywords:** Alzheimer’s disease, Biomarkers, Blood-based biomarkers, Cerebrospinal fluid, Diagnosis, Magnetic resonance imaging, Mild cognitive impairment, Phenotype, Positron emission tomography, Prodromal stage

## Abstract

**Background:**

Development of in vivo biomarkers has shifted the diagnosis of Alzheimer’s disease (AD) from the later dementia stages of disease towards the earlier stages and has introduced the potential for pre-symptomatic diagnosis. The International Working Group recommends that AD diagnosis is restricted in the clinical setting to people with specific AD phenotypes and supportive biomarker findings.

**Main body:**

In this review, we discuss the phenotypic presentation and use of biomarkers for the early diagnosis of typical and atypical AD and describe how this can support clinical decision making, benefit patient communication, and improve the patient journey. Early diagnosis is essential to optimize the benefits of available and emerging treatments. As atypical presentations of AD often mimic other dementias, differential diagnosis can be challenging and can be facilitated using AD biomarkers. However, AD biomarkers alone are not sufficient to confidently diagnose AD or predict disease progression and should be supplementary to clinical assessment to help inform the diagnosis of AD.

**Conclusions:**

Use of AD biomarkers with incorporation of atypical AD phenotypes into diagnostic criteria will allow earlier diagnosis of patients with atypical clinical presentations that otherwise would have been misdiagnosed and treated inappropriately. Early diagnosis is essential to guide informed discussion, appropriate care and support, and individualized treatment. It is hoped that disease-modifying treatments will impact the underlying AD pathology; thus, determining the patient’s AD phenotype will be a critical factor in guiding the therapeutic approach and the assessment of the effects of interventions.

## Introduction

Alzheimer’s disease (AD) is the most common form of dementia, accounting for about two thirds of all cases globally [1]. Worldwide, it is estimated that 41 million individuals with dementia remain undiagnosed, with ~ 25% of all dementia cases clinically identified [2]. A neurodegenerative disorder with various pathobiologic subtypes and clinical presentations [3], AD is defined by neuropathologic changes, including amyloid-beta (Aβ) plaques comprised of aggregated Aβ and neurofibrillary tangles containing aggregated tau proteins [4]. These neuropathologic changes are associated with synapse and neuronal loss, as well as transmitter deficiencies, neuroinflammation, and reactive astrogliosis, which ultimately lead to cognitive impairment [4].

The prototypical clinical phenotype of AD is dementia of insidious onset and gradual progression with prominent amnestic impairment, which represents ~ 85% of cases [5]; however, an important minority of patients with AD pathology present with non-amnestic cognitive impairment [6]. Diagnosis of AD is challenging due to its heterogeneity in pathobiology (e.g., severity, location, and composition), genetic factors, brain resilience, and the resulting distinct clinical presentation (e.g., logopenic variant of primary progressive aphasia [lvPPA], posterior cortical atrophy [PCA], corticobasal syndrome [CBS], and frontal AD). AD pathology can frequently co-occur with other neurodegenerative and vascular diseases, particularly in the aging brain [3]; therefore, differential diagnosis in a timely manner is critical for appropriate care, support, and individualized treatment plans.

The average time between onset of symptoms and diagnosis of AD is approximately 2.8 years [7], and patients may have already progressed into later stages of the disease at the time of diagnosis. There is a spectrum of severity of cognitive impairment in patients with AD, defining different stages in the disease course, including preclinical disease (biomarkers of AD pathology present, but absence of, or subtle, cognitive impairment), prodromal AD (mild cognitive impairment due to AD), mild dementia, and moderate-to-severe dementia due to AD [8].

Early diagnosis is essential to optimize the benefits of available symptomatic medications, which have been shown to alleviate symptoms and delay clinical decline in AD [9]. Moreover, novel disease-modifying treatments (DMTs) for AD are now approved or in development. These are thought to be most effective in the early stages of the disease, and clinical trials have focused on participants with early AD (mild cognitive impairment due to AD or early AD dementia); thus, early diagnosis of AD is even more critical. For example, aducanumab, a monoclonal antibody targeting aggregated Aβ, recently received US Food and Drug Administration approval for the treatment of early AD [10]. In addition, there are several other therapies currently in development for the treatment of AD that may be advanced for clinical use [10].

Development of in vivo biomarkers has shifted the diagnosis of AD from the later dementia stages of disease towards the earlier stages and has introduced the potential for pre-symptomatic diagnosis [11]. Diagnosis of AD, according to the recent recommendations of the International Working Group, is restricted in the clinical setting to people with specific AD phenotypes and supportive biomarker findings [11]. The guidelines state that biomarker-positive cognitively unimpaired individuals should be considered at-risk for progression to AD dementia [11]. A clinical diagnosis is based on the clinical phenotype; biomarkers are becoming increasing available to help distinguish between different disorders and different AD phenotypes, particularly at early stages, and assist in identifying those at risk for symptomatic AD. In this review, we discuss the phenotypic presentation and use of biomarkers for the early diagnosis of typical and atypical AD and describe how this can support clinical decision making, benefit patient communication, and improve the patient journey.

## The clinical phenotypes of AD

**Fig. 1:**
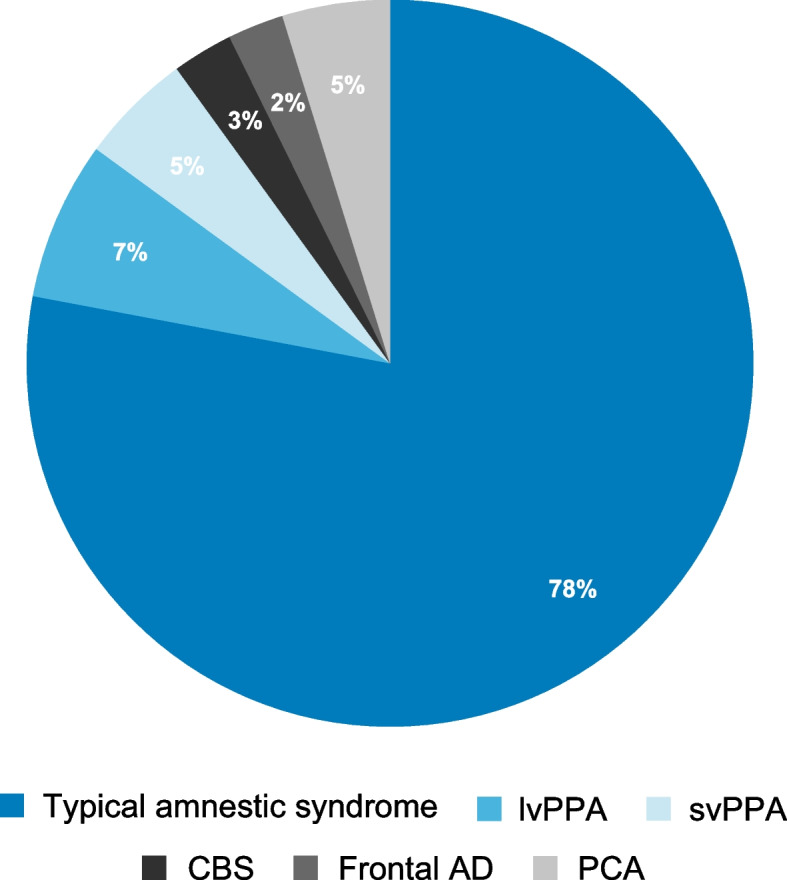
Prevalence of typical and atypical clinical phenotypes of AD in a clinical sample^a^ ^a^The clinical sample comprised 523 patients who were consecutively referred to a specialist dementia clinic and diagnosed with AD; 42 patients had MCI and are not included in the pie chart [12]. Patients with language presentation most likely had lvPPA, though it is possible patients may have had nfPPA. *AD*, Alzheimer’s disease; *CBS*, corticobasal syndrome; *lvPPA*, logopenic variant of primary progressive aphasia; *MCI*, mild cognitive impairment; *nfPPA*, non-fluent primary progressive aphasia; *PCA*, posterior cortical atrophy; *svPPA*, semantic variant primary progressive aphasia

The most typical phenotype of AD is amnestic syndrome (~ 85% of cases) [5]. lvPPA and PCA are less common phenotypes of AD [6]. Other atypical phenotypes include CBS, behavioral or dysexecutive variants of frontal AD, and other variants (e.g., non-fluent primary progressive aphasia [nfPPA] and semantic variant primary progressive aphasia [svPPA]) [6]. The composition of one published clinical sample [12], illustrating the prevalence of typical and atypical clinical phenotypes of AD, is shown (Fig. 1).

Generally, amnestic syndrome is characterized by impairments in learning and recall of newly acquired information [13] and is assessed using semantic cueing (e.g., the Free and Cued Selective Reminding Test [14], which incorporates controlled learning for effective encoding of information and facilitating its retrieval). Patients with amnestic syndrome have poor free recall and decreased total recall (e.g., insufficient efficacy of cueing or impaired recognition) [13], indicating that information is not stored and retrieval is not facilitated.

Patients with lvPPA exhibit deficits in single-word retrieval, sentence repetition, and motor speech abilities (including phonemic paraphasia) [15]. PCA features deterioration of visuospatial and visuoperceptual abilities (with visual inattention or Balint’s syndrome) and impaired arithmetic and reading skills, reflecting the impaired function of the bilateral occipital and parietal cortices, as well as the dorsal and ventral visual streams [16].

CBS is characterized by parkinsonian rigidity, myoclonus, eye and limb apraxia, cortical sensory deficits, and alien limb phenomena [17]. Frontal AD features a progressive clinical syndrome with prominent frontal behavioral features or disproportionate executive dysfunction. More than half of the patients with behavioral and dysexecutive variants of frontal AD present initially with cognitive difficulties more often than with behavioral changes (25% or less), with some cases initially presenting with motor symptoms or a mix of behavioral, cognitive, and/or motor symptoms [18]. Behavioral symptoms include apathy, disinhibition, loss of empathy, and less commonly, perseverative or compulsive behavior, hyperorality, and dietary changes, whereas the dysexecutive variant is dominated by decline in core executive cognitive function, such as working memory and cognitive flexibility [18]. Other primary progressive aphasia phenotypes are less likely to be related to AD pathology: they include nfPPA, which is characterized by poor grammar in written and spoken form with impaired syntactic comprehension and bucco-facial apraxia, and svPPA, notable for problems in comprehending word meanings [15, 19]. Of all lvPPA cases, 86% are due to AD, compared with 20% of nfPPA cases and 16% of svPPA cases [19]. The clinical symptoms associated with the different phenotypes of AD are summarized in Table 1.

**Table 1:** Clinical features of the different phenotypes of AD

Phenotype	Clinical features
Amnestic syndrome [13]	1. Poor free recall 2. Decreased total recall (insufficient efficacy of cueing or impaired recognition) 3. Numerous intrusions
lvPPA [15]	Both of the following core features must be present: 1. Impaired single-word retrieval in spontaneous speech and naming 2. Impaired repetition of sentences and phrases At least three of the following other features must be present: 1. Speech (phonologic) errors in spontaneous speech and naming 2. Spared single-word comprehension and object knowledge 3. Spared motor speech 4. Absence of frank agrammatism
PCA [16]	At least three of the following must be present as early or presenting features ± evidence of their impact on activities of daily living: • Space perception deficit • Simultanagnosia • Object perception deficit • Constructional dyspraxia • Environmental agnosia • Oculomotor apraxia • Dressing apraxia • Optic ataxia • Alexia • Left/right disorientation • Acalculia • Limb apraxia (not limb-kinetic) • Apperceptive prosopagnosia • Agraphia • Homonymous visual field defect • Finger agnosia All of the following must be evident: • Relatively spared anterograde memory function • Relatively spared speech and nonvisual language functions • Relatively spared executive functions • Relatively spared behavior and personality
CBS [17]	• Asymmetric dystonia • Focal or segmental myoclonus • Parkinsonian rigidity • Alien limb phenomena • Limb apraxia • Cortical sensory loss or dyscalculia • Frontal executive dysfunction • Visuospacial deficits
Behavioral variant of frontal AD [18]	• Apathy • Disinhibition • Loss of empathy • Perseverative or compulsive behavior • Hyperorality • Dietary changes
Dysexecutive variant of frontal AD [18]	• Decline in working memory • Decline in cognitive flexibility and inhibition • Absence of behavioral features of frontal AD

*AD* Alzheimer’s disease, *CBS* corticobasal syndrome, *lvPPA* logopenic variant of primary progressive aphasia, *PCA* posterior cortical atrophy

The clinical presentation of AD can be affected by co-pathologies, such as α-synuclein accumulation, vascular pathology, TAR DNA-binding protein 43 pathology, and non-AD tauopathies, particularly in the aging population, as co-pathologies increase with age. The presence of co-pathologies usually leads to a more pronounced appearance of symptoms or variations in the presenting manifestations. As biomarkers are unavailable for the underlying pathology of most non-AD neurodegenerative diseases, separating AD from these diseases depends on identification of a biomarker-supported phenotype or post-mortem examination [11]. Both atypical and typical phenotypes of AD have the canonical biomarkers of this condition; molecular neuroimaging and fluid biomarkers can be used to confirm the AD pathology in vivo.

## Biomarker profiles for typical and atypical phenotypes of AD

The majority of AD biomarkers can be classified into pathophysiologic biomarkers and topographic biomarkers; both can help clinicians to recognize, differentiate, and diagnose AD phenotypes. Pathophysiologic biomarkers related to AD lesions include amyloid positron emission tomography (PET), cerebrospinal fluid (CSF) concentrations of amyloid and tau proteins, and plasma concentrations of amyloid, tau, and other protein biomarkers. Topographic biomarkers are related to the regional consequences of AD pathology, such as regional hypometabolism on fluorodeoxyglucose (FDG)-PET, tau PET, and regional/local atrophy on structural magnetic resonance imaging (MRI).

**Fig. 2:**
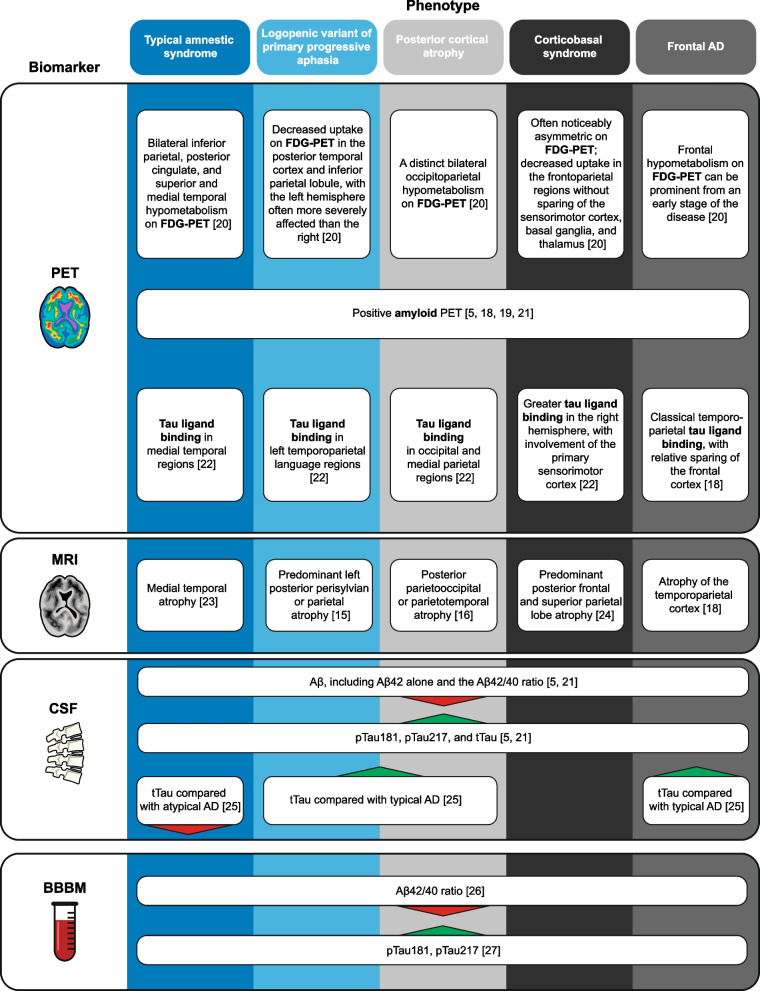
Summary of anticipated biomarker results for the different clinical phenotypes of AD. Decreased plasma Aβ42/40 ratio is extended across all clinical phenotypes, as there is evidence of increased Aβ plaques across the phenotypes. Decreased plasma Aβ42/40 ratio has been identified across the AD continuum [26, 28]; however, further studies are warranted to investigate the utility of the Aβ42/40 ratio across the clinical phenotypes. *Aβ*, amyloid-beta; *Aβ42/40* Aβ (1–42)/(1–40); *AD*, Alzheimer’s disease; *BBBM*, blood-based biomarker; *CSF*, cerebrospinal fluid; *FDG*, fluorodeoxyglucose; *FTD*, frontotemporal dementia; *MRI*, magnetic resonance imaging; *PET*, positron emission tomography; *pTau*, phosphorylated tau; *tTau*, total tau

To date, no fluid biomarkers have been conclusively linked to an individual clinical phenotype of AD; however, according to their regional distribution differences, there are corresponding molecular, metabolic, and degenerative imaging biomarkers for the different phenotypes of AD (Fig. 2) [5, 15, 16, 18–27]. The existing literature is predominantly focused on biomarkers of AD pathology, where the main clinical value of their uses is to discriminate between phenotypes that are associated with AD vs. non-AD pathologies, and to measure the advances in underlying biology such as plasma and CSF levels of phosphorylated tau (pTau). Currently, there is no single biomarker or biomarker algorithm that can differentiate between AD phenotypes with conclusive diagnostic accuracy, underscoring the importance of clinical evaluation.

## PET markers

**Fig. 3:**
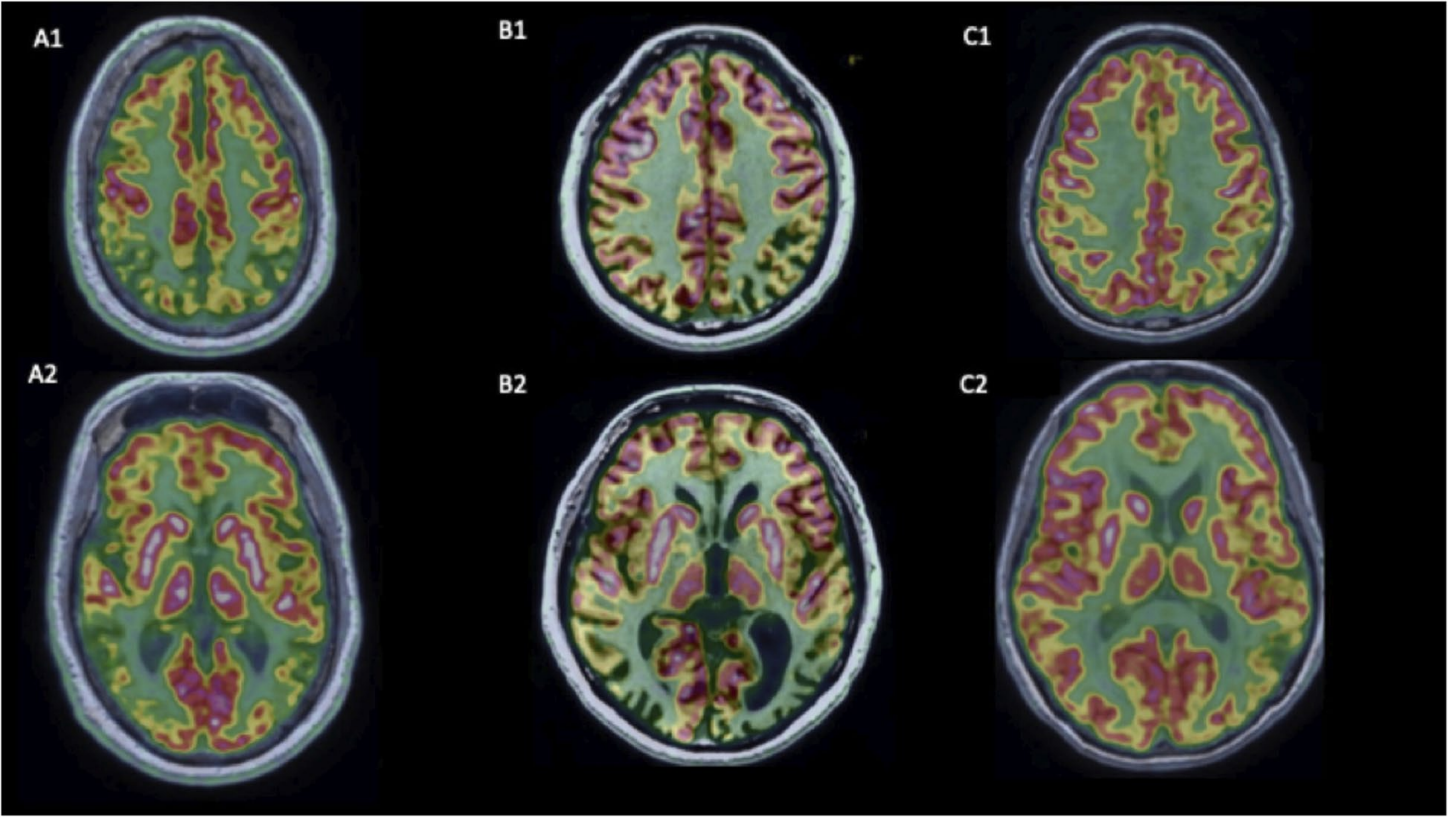
FDG-PET across the clinical phenotypes of AD: **A** typical AD, **B** PCA, and **C** lvPPA. Panel **A** shows symmetrical bilateral parietal and temporal hypometabolism in a patient with typical AD. Panel **B** shows bilateral reduction in parieto-tempo-occipital metabolism in a patient with PCA. Panel **C** shows greater hypometabolism in the left parieto-temporal region compared with the right parieto-temporal region in a patient with lvPPA. Panels **1** and **2** show different depths within the brain. The FDG-PET images in Fig. 3 are courtesy of Dr. Marie-Odile Habert (Department of Nuclear Medicine, Pitié-Salpêtrière Hospital, APHP, Paris). *AD*, Alzheimer’s disease; *FDG*, fluorodeoxyglucose; *lvPPA*, logopenic variant of primary progressive aphasia; *PCA*, posterior cortical atrophy; *PET*, positron emission tomography

PET imaging with a variety of ligands can help clinicians to identify the underlying pathology of typical and atypical phenotypes of AD and assist in the differential diagnosis of other neurodegenerative diseases. Examples of PET imaging across the clinical phenotypes of AD are shown (Fig. 3).

FDG-PET is a topographic biomarker which can help to characterize typical and atypical AD, with patterns of regional hypometabolism reflecting clinical deficits across AD variants [29]. Patients with AD typically exhibit hypometabolism bilaterally in the parietal and medial temporal regions, including the precuneus [20]. This imaging technique can also detect patterns of hypometabolism characteristic of non-AD dementias, such as dementia with Lewy bodies and frontotemporal dementia (FTD), thus enabling differential diagnosis [20]. While FDG-PET provides greater diagnostic information than MRI, it is not as widely used.

Amyloid PET is predominantly used in the research setting and visualizes fibrillar or insoluble Aβ plaques in the brain, but not other forms of the Aβ peptide [30]. Amyloid PET is the most extensively validated biomarker for the identification of amyloid plaques, which has demonstrated high accuracy in imaging-to-autopsy studies (sensitivity, 92%; specificity, 100%) [31]. Unlike other imaging modalities, amyloid distribution as visualized by PET is similar in patients with atypical and typical AD [32].

Tau PET, which visualizes neurofibrillary tangles, is predominantly used in the research setting; it is the only autopsy-validated (sensitivity, 92 − 100%; specificity, 52 − 92%), direct marker of neurofibrillary tangles, as fluid markers have not demonstrated similar accuracy [33]. Compared to amyloid PET, tau PET has been shown to predict cognitive decline in cognitively unimpaired individuals and is of high clinical relevance as assessment is associated with short-term progression (3 − 5 years) in these individuals [34, 35]. In contrast to amyloid PET, tau PET is a topographic technique and deposition patterns are more reflective of the clinical phenotype [22]. Tau PET ligand binding has been shown to be greater in occipital regions in posterior cortical atrophy, left frontal regions in logopenic aphasia, and medial temporal areas in patients with the amnestic form of AD [22]. Tau PET patterns differ between typical (amnestic form) and atypical AD, and these differences might be useful in characterizing the biology corresponding to typical and atypical phenotypes.

Other types of PET biomarkers currently under investigation to aid diagnosis of AD include synaptic vesicle glycoprotein 2A PET for synaptic density and translocator protein PET for neuroinflammation [36, 37]. Of note, these biomarkers would play a role in disease staging and prognostication, not differential diagnoses. Additionally, these PET tracers are hardly used in clinical practice.

## MRI markers

MRI changes are topographic biomarkers of AD, and structural MRI in patients with AD shows atrophy of grey matter and volume loss, indicating neurodegeneration [30]. Serial structural MRI imaging is often used to measure disease progression as this approach has lower variance than single structural MRI [38]. Some data indicate that serial structural MRI may have the potential to evaluate therapeutic efficacy in clinical practice, as serial structural MRI measurements could permit clinical trials to be performed with smaller sample sizes than would be possible using traditional clinical assessments [38]. There are additional MRI measures such as: diffusion tensor imaging MRI, to assess damage to white matter [39]; resting state functional MRI, to assess changes in functional brain connectivity that are thought to precede structural brain changes [40]; and T2-weighted or susceptibility-weighted MRI, to assess vascular activity and identify cerebral amyloid angiopathy [41]. However, these techniques are not currently used in the majority of clinical trials or in routine clinical practice but may become increasingly important to manage amyloid-related imaging abnormalities (ARIA) with the use of anti-amyloids.

On structural MRI, atrophy in the typical form of AD begins in the medial-temporal lobe and progresses to involve the lateral-temporal and parietal cortices, whereas in atypical AD, atrophy is usually most prominent in regions corresponding to clinical symptoms and is often hippocampus-sparing in early-stage disease [29]. Alterations in MRI correspond to those seen in FDG-PET but usually appear later in disease progression. Free and Cued Selective Reminding Test memory scores of patients with amnestic syndrome have been shown to correlate with hippocampal volume assessed with structural MRI in patients with AD [13].

## CSF biomarkers

CSF biomarkers can be used to detect both Aβ and tau peptides and, in most patients, alterations in these biomarkers can be detected earlier than changes in neuroimaging biomarkers [30]. CSF tau indicates neuronal death, and, therefore, it can be elevated in atypical phenotypes as well as in non-AD dementias; an increase in pTau is more specific to AD pathology in both typical and atypical presentations [25, 42]. CSF biomarkers include Aβ (1–42) (Aβ42), Aβ (1–40) (Aβ40), pTau 181 (pTau181), pTau 217 (pTau217), and total tau (tTau). These markers have been shown to accurately identify AD-associated biologic changes, even at pre-symptomatic and prodromal stages of disease progression, and some have demonstrated the potential to predict cognitive decline [43].

CSF Aβ42 concentration and Aβ42/40 ratio decrease and correlate inversely with the extent of Aβ plaque deposition [44], and concentrations of tTau and pTau correlate with the intensity of neurodegeneration and neurofibrillary tangles, respectively [45]. The presence of a decreased CSF Aβ42/40 ratio typically indicates AD pathology, even in those with atypical clinical features or irregular CSF findings for other analytes [46]. With respect to a “FTD-phenotype,” the pTau181/Aβ42 ratio is useful for the identification of both the behavioral and semantic variants of frontal AD and in differentiating these patients from those with frontotemporal dementia [47]. CSF pTau 231 (pTau231) is another pTau epitope that has demonstrated the potential to aid in diagnosis of early AD and detect incipient AD pathology, not confined to the clinical phenotype [48].

In some clinics, neurofilament light chain (NfL) has replaced tTau as a biofluid measure of neurodegeneration due to its high sensitivity [30]. Other CSF biomarkers currently under investigation to aid in AD diagnosis include chitinase 3-like 1 (also known as YKL-40), glial fibrillary acidic protein (GFAP), and neurogranin and other synaptic biomarkers [49–52]. Of note, these biomarkers would play a role in disease staging and prognostication, not differential diagnoses.

## Blood-based biomarkers (BBBMs)

BBBMs are emerging as important tools to aid in identification of pathology associated with AD; concentrations of Aβ peptides and pTau in the blood have demonstrated associations with the corresponding concentrations in CSF and with PET positivity [53]. Assays that measure the plasma Aβ42/40 ratio are commercially available in the US and have shown potential in determining Aβ positivity at all stages of the AD continuum [28]. Further validation of such assays is required, particularly to understand whether the plasma Aβ42/40 ratio would be sufficiently scalable to provide timely support for clinical diagnosis, as there are potential issues with robustness [28]. Plasma pTau181, pTau217, and pTau231 have shown promise in detecting advancing tau-related AD pathology [27, 48]. In addition, blood GFAP has demonstrated the potential to predict cognitive decline to AD dementia in patients with mild cognitive impairment [54] and to differentiate FTD from AD [55].

One study has demonstrated that NfL is increased in the serum of patients with primary progressive aphasia compared with controls and discriminates between nfPPA/svPPA and lvPPA with 81% sensitivity and 67% specificity [56]; while plasma concentrations of Aβ peptides and pTau may be more accurate in differentiating AD from other diagnoses, NfL has the potential to support the diagnosis of AD-related primary progressive aphasia, particularly lvPPA. In the future, it is likely that BBBMs will be a useful triaging tool in the detection of AD, ensuring that appropriate patients receive neurocognitive assessment and confirmatory biomarker testing in a timely manner. Biomarker profiles cannot fully replace clinical assessment; thus, BBBMs are more likely to become a tool used in conjunction with clinical history and imaging to support clinical decision making.

Apolipoprotein E (APOE) is a blood-based genetic biomarker, and the APOE ε4 genotype is a risk factor for AD. One of the factors that can increase the risk of progression to symptomatic AD is APOE ε4 gene carrier status, with APOE ε4 homozygotes being at very high risk of developing clinical AD [11]. Thus, the presence of APOE ε4 in an individual with symptoms consistent with AD supports the likely occurrence of amyloid pathology. The use of polygenic risk scores which exclude the APOE region (to determine the contribution of non-APOE genes) combined with APOE status may provide the best strategy to identify individuals at increased risk of dementia [57]. Regarding clinical phenotypes, the typical amnestic phenotype is promoted by the APOE ε4 allele, whereas the atypical non-memory phenotypes often occur in the absence of the APOE ε4 allele [58].

## Biomarkers should be complementary to clinical assessment for AD diagnosis

Traditionally, AD diagnosis was based solely on clinical criteria; however, current guidelines for AD diagnosis also consider the presence of biologic markers. For the neuropathologic diagnosis of AD using biomarkers, the National Institute on Aging and Alzheimer's Association (NIA-AA) “ATN” research framework can be used [23]. This framework proposes to define AD based on amyloid abnormalities (‘A’), tau protein changes (‘T’), and evidence of neurodegeneration (‘N’), irrespective of clinical phenotypes and even in the absence of cognitive symptoms. The ATN research framework guidelines conclude that neuropathologists can certify the presence of AD pathology in the brain, yet they cannot diagnose the clinicopathologic syndrome recognized as AD [23].

The intended use of the NIA-AA “ATN” research framework is for research purposes [23]; therefore, there is debate around the use of this framework in clinical practice. Biomarkers alone are not sufficient to inform AD diagnosis and should be used as a supplement to clinical assessment to support or confirm the clinical diagnosis [11]. Although tau PET has been shown to predict cognitive decline in cognitively unimpaired individuals, biomarker-only diagnosis of AD generally has modest predictive accuracy in cognitively unimpaired individuals [11] because these subjects may show amyloid positivity and not display clinical manifestations within their lifetime. The current estimates of lifetime dementia risk in cognitively unimpaired individuals with amyloidosis range from 44–74% [59]. It is important to note that tau PET and CSF measures of tau are not directly comparable, neither biologically nor in terms of clinical progression [60]. As noted above, the presence of tau changes is generally an indicator of more rapid progression. Moreover, without clinical assessment or without clinical symptoms, AD diagnosis based on biomarkers alone may be harmful to a patient without counseling. Patients should be aware that a positive biomarker result identifies an AD risk state and there is a possibility that AD symptoms may not manifest within their lifetime; it is important that patients understand the varied nature and progression of AD. Patients with clinically diagnosed AD may not show biomarker positivity. Up to 35% of patients with clinically diagnosed AD have shown amyloid PET negativity [61], indicating that the AD phenotype is not always indicative of AD pathology in the brain.

Another major limitation of biomarker-only AD diagnosis is the high variability in disease progression among individuals with biomarker-positivity. The risk of progression depends on several factors and can be related to multiple mechanisms, such as brain resistance (i.e., observed absent or low levels of pathology despite substantial AD risk factors), resilience (i.e., remaining cognitively normal despite high levels of AD pathology), and reserve (i.e., physiologic pre-morbid capacity) [62]. A substantial proportion of people remain cognitively normal throughout their lifetime, showing AD pathology, such as amyloidosis, only at autopsy or on vivo imaging (~ 30%) [62].

Many dementias have a mixed etiology and identifying non-AD dementias is challenging given that biomarkers for non-AD pathologic changes are mostly unavailable. Differential diagnosis largely depends on phenotype or post-mortem examination. Even among experts, the diagnostic accuracy for a clinical determination of AD is about 75–80% [63]. However, CSF biomarkers alone (Aβ42, pTau181, and tTau) can discriminate between non-AD dementias (including dementia with Lewy bodies, FTD, vascular dementia, and Parkinson’s disease dementia) and AD with a diagnostic accuracy of 82.7%, which is comparable to the clinical diagnostic accuracy of 81.6%, based on a thorough clinical work-up including neuroimaging [64]. Moreover, amyloid PET and tau PET have demonstrated the ability to differentiate between AD and frontotemporal lobar degeneration [65, 66]. Amyloid and tau PET are positive in AD and negative in non-AD disorders. The potential differential diagnoses of the different phenotypes of AD are summarized in Table 2.

**Table 2:** Potential differential diagnoses of the different phenotypes of AD

Phenotype	Potential differential diagnoses
Amnestic syndrome [11, 20, 67]	• Limbic-predominant age-related TDP-43 encephalopathy • Dementia with Lewy bodies • Primary age-related tauopathy • Frontotemporal lobar degeneration
lvPPA [68]	• Mild cognitive impairment • nfPPA • svPPA
PCA [16, 20]	• Dementia with Lewy bodies • Cortical basal degeneration • Prion diseases, such as Creutzfeldt-Jakob disease
CBS [21]	• Cortical basal degeneration • Progressive supranuclear palsy • Frontotemporal lobar degeneration
Behavioral variant of frontal AD [20]	• Behavioral variant of FTD
Dysexecutive variant of frontal AD [69]	• Behavioral variant of FTD

*AD* Alzheimer’s disease, *CBS* corticobasal syndrome, *FTD* frontotemporal dementia, *lvPPA* logopenic variant of primary progressive aphasia, *nfPPA* non-fluent primary progressive aphasia, *PCA* posterior cortical atrophy, *svPPA* semantic variant primary progressive aphasia, *TDP-43* TAR DNA-binding protein 43

It is important to note that the “ATN” research framework is a limited repertoire of biomarkers to be considered in the diagnosis of AD. Other biomarkers that warrant consideration for inclusion in the framework include NfL (neurodegeneration), beta-synuclein (synaptic degeneration), GFAP (astrogliosis), and inflammatory biomarkers [30].

## Biomarkers to support clinical decision making

**Fig. 4:**
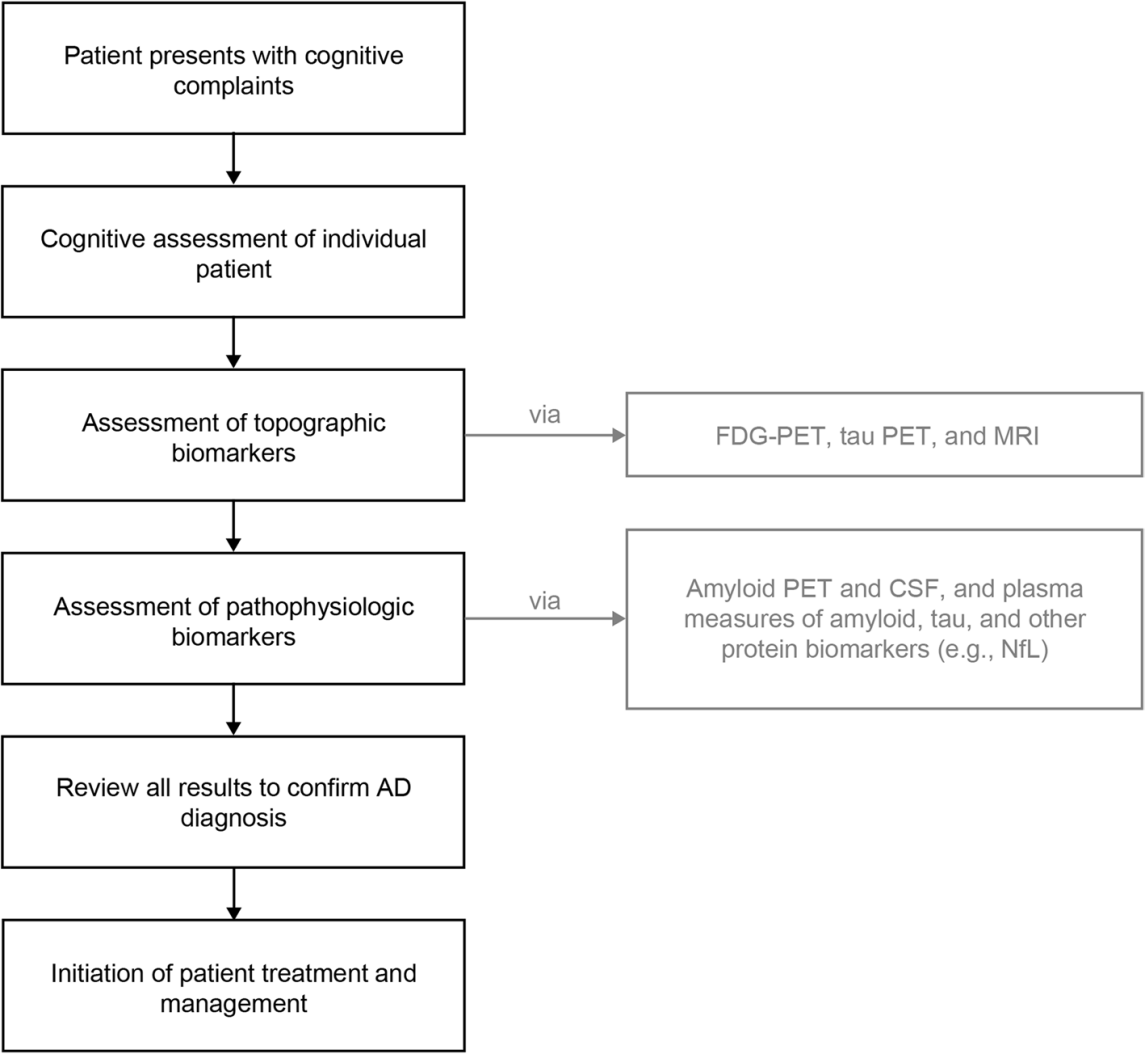
The role of AD biomarkers in the clinical setting. *AD*, Alzheimer’s disease; *CSF*, cerebrospinal fluid; *FDG*, fluorodeoxyglucose; *MRI*, magnetic resonance imaging; *NfL*, neurofilament light chain; *PET*, positron emission tomography

AD biomarkers play a confirmatory role in clinical decision making, which is particularly important with the advancement of anti-Aβ DMTs (Fig. 4). These DMTs aim to delay the onset or slow the progression of cognitive changes and, thus, earlier intervention in the disease course is desirable. By identifying the underlying AD pathology, biomarkers can aid early diagnosis and have the potential to identity patients at risk of AD, in some cases before clinical presentation [70]. AD biomarkers established in clinical practice include MRI, FDG-PET, tau PET, and CSF measures of amyloid and tau, as well as plasma biomarkers, which are well on the way to being approved.

The informed use of biomarkers in the clinical setting promises to assist in drug development for patients with and at risk of AD. For example, APOE ε4 homozygotes present a high-risk population for research and clinical trials for preclinical AD [70]. Though it is unlikely that APOE ε4 status will have a pivotal role in the clinical diagnosis of AD, APOE ε4 assessment may be significant with the arrival of anti-Aβ DMTs, as APOE ε4 carriers have higher incidence of ARIA [71]. Amyloid PET is a quantitative measure of amyloid plaque burden used to aid in diagnosis of AD; it provides efficient and objective patient classification for research and clinical trials but may be less useful in clinical practice because of cost and lack of availability. Plasma pTau181 may be valuable for clinical trials as recent studies have demonstrated that this biomarker accurately predicts the transformation to symptomatic AD in individuals without cognitive impairment, with reasonable sensitivity and accuracy [72]. Plasma pTau217 may be useful for participant selection in clinical trials, as well as for disease monitoring [73]. Moreover, FDG-PET may represent a more widely available tool to predict the progression of AD in patients with mild cognitive impairment and could be used to obtain an estimate of the time free from disease progression [74].

Biomarkers facilitate the improved detection of both amnestic and non-amnestic AD phenotypes in vivo. Patients presenting with atypical AD clinical syndromes often receive a later diagnosis than those presenting with typical AD [29]. One study showed that in patients with young-onset AD, incorrect diagnoses were common in individuals with atypical presentations (53%) compared with patients with typical presentations (4%) [75]. The availability of AD biomarkers and the incorporation of atypical AD phenotypes into diagnostic algorithms allow these patients to be more confidently identified and diagnosed earlier in the disease course and, therefore, to be offered tailored information, appropriate care and support, and construction of individualized treatment plans [9]. There is value in informing patients and their families of their prognosis, as patients can learn how to adjust to the diagnosis and, based on the available information, can make choices and plans [9]. These advances will provide improved access to clinical trials for DMTs, which often exclude patients with atypical phenotypes.

## Conclusions

Atypical presentations of AD often mimic other dementia sub-types and differential diagnosis can be facilitated using AD biomarkers. AD biomarkers alone are not sufficient to confidently diagnose AD or predict progression to AD dementia; biomarkers should be supplementary to clinical assessment to help inform the diagnosis of AD. Without clinical assessment, AD biomarkers alone may be harmful when results are presented to a patient without counseling; patients should understand the varied nature and progression of AD and the possibility that a positive biomarker result in asymptomatic individuals may not predict the occurrence of AD symptoms within their lifetime. Use of AD biomarkers and improved recognition of atypical AD phenotypes into diagnostic criteria will allow for the earlier diagnosis of patients with atypical clinical presentations that otherwise would have been misdiagnosed and treated inappropriately. Early diagnosis is essential to guide tailored information, appropriate care and support, and individualized treatment. As it is hoped that DMTs will impact the underlying AD pathology, characterizing the patient’s AD phenotype will be a critical factor in guiding the therapeutic approach and the assessment of the effects of interventions.

## Data Availability

Not applicable.

## Abbreviations

Aβ: Amyloid-beta
Aβ40: Amyloid-beta (1–40)
Aβ42: Amyloid-beta (1–42)
AD: Alzheimer’s disease
APOE: Apolipoprotein E
APOE ε4: Apolipoprotein E ε4 genotype
ATN: Amyloid plaques (‘A’), neurofibrillary tau tangles (‘T’), and neurodegeneration (‘N’)
BBBM: Blood-based biomarkers
CBS: Corticobasal syndrome
CSF: Cerebrospinal fluid
DMT: Disease-modifying treatment
FDG: Fluorodeoxyglucose
FTD: Frontotemporal dementia
GFAP: Glial fibrillary acidic protein
lvPPA: Logopenic variant of primary progressive aphasia
MRI: Magnetic resonance imaging
NIA-AA: National Institute on Aging and Alzheimer's Association
NfL: Neurofilament light chain
nfPPA: Non-fluent primary progressive aphasia
PCA: Posterior cortical atrophy
PET: Positron emission tomography
pTau: Phosphorylated tau
svPPA: Semantic variant primary progressive aphasia
tTau: Total Tau
YKL-40: Chitinase 3-like 1
